# The dependence of particle size on cell toxicity for modern mining dust

**DOI:** 10.1038/s41598-023-31215-5

**Published:** 2023-03-29

**Authors:** Yi-Hsuan Chen, Dorothy Nguyen, Stephen Brindley, Tiancong Ma, Tian Xia, Jürgen Brune, Jared M. Brown, Candace Su-Jung Tsai

**Affiliations:** 1 Department of Environmental Health Sciences, Fielding School of Public Health, University of California, Los Angeles, 650 Charles E. Young Drive S., MC 177220, Los Angeles, CA 90095-1735 USA; 2 Department of Environmental and Occupational Health, University of Colorado Anschutz Medical Campus, Aurora, CO USA; 3 Department of Medicine, California NanoSystems Institute, University of California, Los Angeles, Los Angeles, CA USA; 4 Mining Engineering Department, Colorado School of Mines, Golden, CO USA; 5 Department of Pharmaceutical Sciences, University of Colorado Anschutz Medical Campus, Aurora, CO USA

**Keywords:** Cell biology, Stem cells, Biomarkers, Health occupations, Nanoscience and technology

## Abstract

Progressive massive pulmonary fibrosis among coal miners has unexpectedly increased. It would likely due to the greater generation of smaller rock and coal particles produced by powerful equipment used in modern mines. There is limited understanding of the relationship between micro- or nanoparticles with pulmonary toxicity. This study aims to determine whether the size and chemical characteristics of typical coal-mining dust contribute to cellular toxicity. Size range, surface features, morphology, and elemental composition of coal and rock dust from modern mines were characterized. Human macrophages and bronchial tracheal epithelial cells were exposed to mining dust of three sub- micrometer and micrometer size ranges at varying concentrations, then assessed for cell viability and inflammatory cytokine expression. Coal had smaller hydrodynamic size (180–3000 nm) compared to rock (495–2160 nm) in their separated size fractions, more hydrophobicity, less surface charge, and consisted of more known toxic trace elements (Si, Pt, Fe, Al, Co). Larger particle size had a negative association with in-vitro toxicity in macrophages (*p* < 0.05). Fine particle fraction, approximately 200 nm for coal and 500 nm for rock particles, explicitly induced stronger inflammatory reactions than their coarser counterparts. Future work will study additional toxicity endpoints to further elucidate the molecular mechanism causing pulmonary toxicity and determine a dose–response curve.

## Introduction

Respirable coal mine dust is a well-known cause of numerous negative health effects. Coal mine dust lung diseases (CMDLD) include coal workers’ pneumoconiosis (CWP), silicosis, progressive massive fibrosis (PMF), chronic dust-related diffuse fibrosis (DDF) and chronic obstructive pulmonary disease (COPD)^1,2^. Implementation of permissible exposure limits (PEL) and control technologies contributed to a temporary decline in progressive pulmonary diseases from the mid-1970s to the late 1990s^3^. Surprisingly, there has been a resurgence of CMDLD since the year 2000^4^, likely due to the widespread utilization of more powerful and larger-scale, electric or diesel-powered mining equipment generating more toxic small particles^5,6^. The National Institute for Occupational Safety and Health (NIOSH) determined that these diseases were more pronounced in miners with longer tenure in underground coal mines. The Coal Workers’ Health Surveillance Program (CWHSP) at NIOSH is also concerned about the acceleration of disease progression^2,7^. The urgent need to understand dust exposure in modern mines with productive mining devices is also emphasized by the elevation of coal consumption, which will remain high for decades, making coal an irreplaceable energy source worldwide^8^.

The respiratory system is the first deposition position for inhaled fine particles. The size and unique surface properties of the particles contribute to pulmonary deposition and systemic translocation. While larger particles typically deposit in the upper respiratory tract and in the airways to a lesser extent, ultrafine particles are more capable of penetrating even deeper lung regions like the alveoli and terminal bronchioles^6,9–11^. Physical contact with harmful particles can directly induce toxicological responses in lung cells, including necrosis, apoptosis, or cell death. To eliminate these foreign substances, lung bronchiolar epithelial and immune cells are activated and initiate phagocytosis. The clearance process produces various cytokines including interleukin-1 (IL-1) and tumor necrosis factor-alpha (TNF-α) that promote the inflammatory response^12–14^. Lung macrophage activation also increases the production of reactive oxygen species (ROS) leading to oxidative stress^15,16^. Cytokines and extracellular matrices released in response to continuous coal exposure stimulate the formation of fibroblasts and subsequent fibrosis^17^. The mechanistic pathways responsible for fibrotic CMDLDs are complicated, leading to the challenge of elucidating the role of fine particles in toxicity activation.

Higher-powered equipment in modern mines generates finer and greater amounts of airborne particles^5,6^ and is a highly suspected cause of the increase in lung disease prevalence in recent years. This equipment is powerful enough to cut rock and is often used to excavate higher spaces underground where coal beds are getting progressively thinner. Particle size alone does not determine biological transformation and responses; surface properties, morphology, elemental composition, agglomeration, and hydrophobicity or hydrophilicity are also critical in toxicokinetic^16,18^. Occupational pulmonary toxicity is strongly related to heavy metal and crystalline silica exposure^19,20^. In fact, anthracite coal miners have a higher rate of pneumoconiosis than non-anthracite miners, which is suspected to be associated with the higher silica content in anthracite coal dust^21^.

Most studies regarding particle characteristics and molecular biological responses focus on engineered materials or ambient particulate matter^22–24^, with very limited data on airborne coal mining dust. The characteristics of mining dust particles that contribute to occupational lung diseases among miners remain unclear. The present study aims to determine whether the submicron size fraction of mining dust contributes to pulmonary cellular toxicity and uses human cell culture models to identify factors contributing to cellular toxicity. Coal dust is released when coal is cut into. Rock dust is pure limestone not natural present in coal mine. However, large amounts of rock dust are brought in to smother coal in order to prevent explosions. To support the hypotheses that coal and rock respirable dust have different properties contributing to cell toxicity, the following objectives were achieved: (1) coal and rock dust particles were initially separated by particle size and characterized via metric approaches to observe their size range, surface features, morphology, and elemental composition; (2) human macrophages and bronchial tracheal epithelial cells (HBEC’s) were exposed to dust particles, and examined for cytotoxicity and inflammation; (3) the effect of size on cell responses was evaluated.

## Results

### Dispersibility, size range, and morphology of dust samples

Rock and coal dust solutions of 1 mg/ml in water showed visibly different suspension dispersibility (Fig. S1). After 10 min water bath sonication, the unseparated rock particles quickly settled at the bottom and the unseparated coal particles remained suspended in the solution. After the separation of dust samples, dynamic light scattering (DLS) analysis measured the size distribution in the dust suspension (Fig. S2). Coal had hydrodynamic size peaks for the top layer at 188 nm and 1130 nm (named C_0.2_), and for the coal middle layer at 767 and 838 nm (C_0.7_) as measured by DLS. Following separation, visibly large coal bottom layer particles settled to the bottom of the DLS cuvette quickly. Due to the limitation, the size of coal particles from the bottom layer was determined by transmission electron microscopy (TEM) to range from 1000 to 3000 nm (named C_1_). The centrifugation method of rock had peaks for the top layer at 495 nm (named R_0.5_), for the rock middle layer at 1110 nm (R_1_), and for the rock bottom layer at 1790 nm and 2160 nm (R_2_) measured by DLS. All rock layer sizes measured by DLS were consistent with TEM images. DLS and TEM sizing of coal particles was consistent for 5 of the 6 separations, all but the C_1_. The rapid deposition observed only in C_1_ that necessitated TEM sizing may be due to its largest size fraction (up to 3000 nm, Table 1) and more hydrophobic characteristics.

**Table 1:** Summary of respirable mining dust particles characteristics.

Sample	Z Potential in water (mV)	Hydrodynamic size peak (nm)	Elements (wt%)^1^	Functional groups present	Contact angle (°)
Coal	− 17.10 ± 1.23	570	C (90.2%), O (4.4%), Cu (2.9%), Si (1.3%), Al (1.0%), Fe, Ca, Ba, Cl, K, Pt (0.0%)	–OH, CH, C=O, C=C	86° ± 5
C_0.2_	− 21.70 ± 1.92	188, 1130			
C_0.7_	− 25.03 ± 0.21	767, 838			
C_1_	− 24.50 ± 0.53	1000–3000			
Rock	− 38.70 ± 1.94	520	C (61.4%), O (14.5%), Ca (13.5%), Cu (9.5%), Fe (0.5%), Co (0.3%), Mn (0.2%), Si (0.1%)	–OH, CH, pyran ring	NA
R_0.5_	− 19.40 ± 0.54	495			
R_1_	− 14.60 ± 0.30	1110			
R_2_	− 7.83 ± 1.06	1792, 2155			

**Figure 1:**
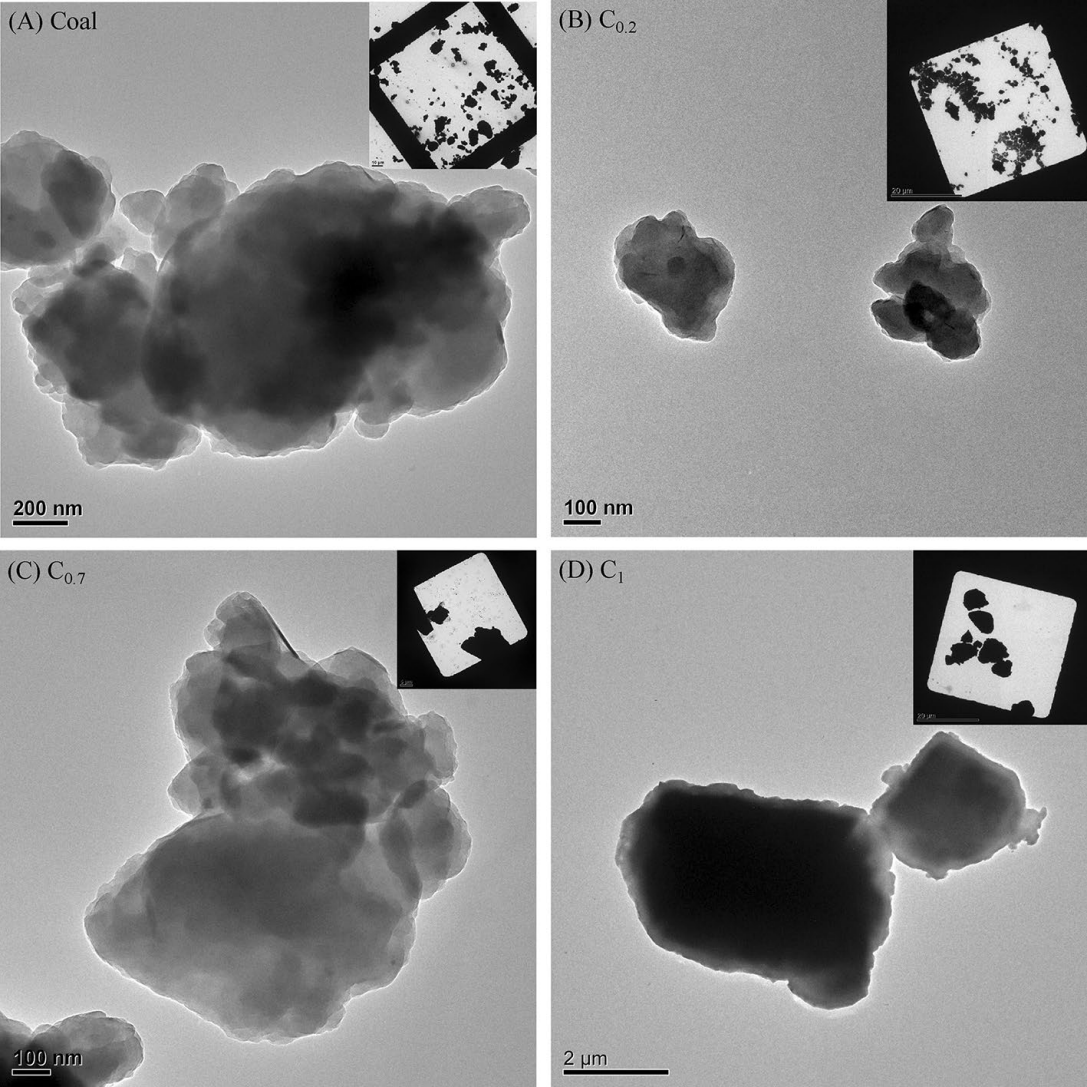
TEM square view and individual particles images. (**A**) Raw coal; (**B**) C_0.2_: coal top layer; (**C**) C_0.7_: coal middle layer; and (**D**) C_1_: coal bottom layer from coal suspension.

**Figure 2:**
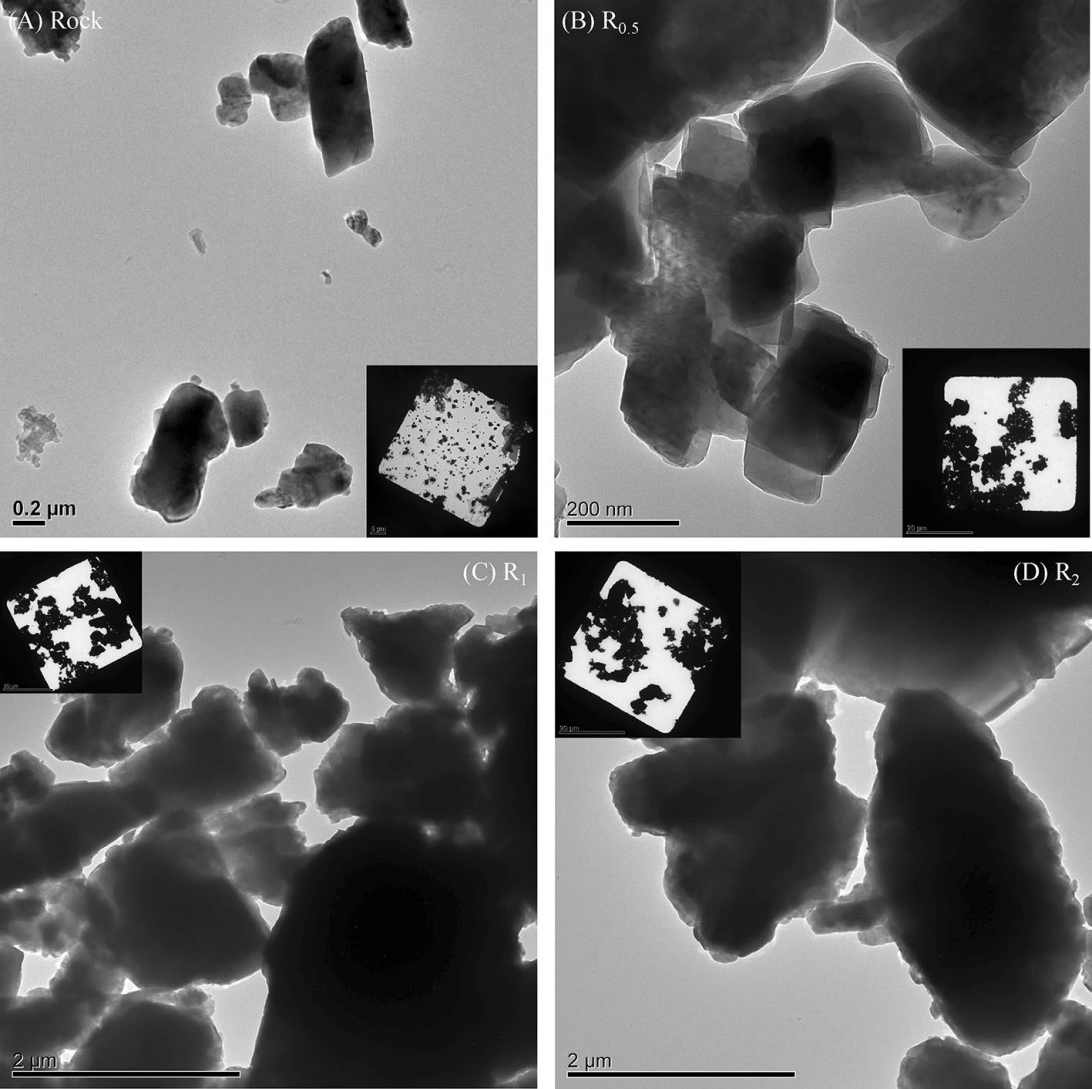
TEM square view and individual particles images. (**A**) Raw rock; (**B**) R_0.5_: rock top layer; (**C**) R_1_: rock middle layer; and (**D**) R_2_: rock bottom layer from rock suspension.

The high-magnification microscopy images revealed the morphology of dust particles and confirmed successful size separation as shown in Figs. 1 and 2. Both raw, unseparated coal and rock dust images included particles smaller than 200 nm and coarse particles around 600 nm, consistent with the hydrodynamic size distribution measured by DLS. Identically, the coal particles in the top layer were around 200–300 nm in the TEM images, which were finer than the rock dust. The size difference between the middle and bottom layers is apparent. The coal particles (Fig. 1) were rounder than the rock (Fig. 2), which appeared sharp in the images. In summary, the dispersibility and morphology results differ between coal and rock dust.

### Surface features of raw coal and rock dust

The contact angle is related to the hydrophobicity of a substance. The contact angle of raw coal and rock dust was measured immediately upon contact with the water droplet. This was repeated three times. The contact angle of raw coal dust was around 86° $\pm$ 5° (Fig. S3). In contrast, a contact angle could not be determined for rock because the droplet immediately dispersed over the surface of the rock pellet, indicating higher hydrophilicity. The contact angle measurement indicate that coal is more hydrophobic than rock.

### Elemental composition for raw coal and rock dusts

Elemental composition percentages from scanning transmission electron microscopy with energy dispersive X-ray (STEM w/ EDX) are shown in Table 1 and the spectrum map is displayed in Fig. S4. The spectrum showed that the rock dust, mostly limestone, consisted of carbon (C; 61.4%), oxygen (O; 14.5%), and calcium (Ca; 13.5%). There was some copper (Cu; 9.5%), iron (Fe; 0.5%), cobalt (Co; 0.3%), manganese (Mn; 0.2%), and silicon (Si; 0.1%). Coal dust had carbon (C; 90.2%), oxygen (O; 4.4%), copper (Cu; 2.9%), silicon (Si; 1.3%), and aluminum (Al; 1.4%).

Functional groups were determined using Fourier-transform infrared spectroscopy (FTIR). While both dusts contain hydroxyl and aliphatic groups, coal’s hydrophobic CH_3_ and CH_2_ groups indicated in the blue 2850–293 cm^−1^ region had a stronger signal compared to coal’s hydrophilic -OH stretching indicated in the red 3200–3600 cm^−1^ region^25^ compared to that of rock (Fig. S5). Rock has less prominent CH_3_ and CH_2_ strength relative to the corresponding –OH stretching. Furthermore, as seen in Fig. S5, an aromatic C=C peak at 1600 cm^−1^ was present in coal but not in rock. Rock has characteristic calcite peaks. The zeta potential given in Table 1 ranges from − 38.70 to − 7.83 mV. Bulk rock appeared to be more stable as determined by zeta potential compared to size fraction rock.

### Toxicity induced by dust particles dependent on size

**Figure 3:**
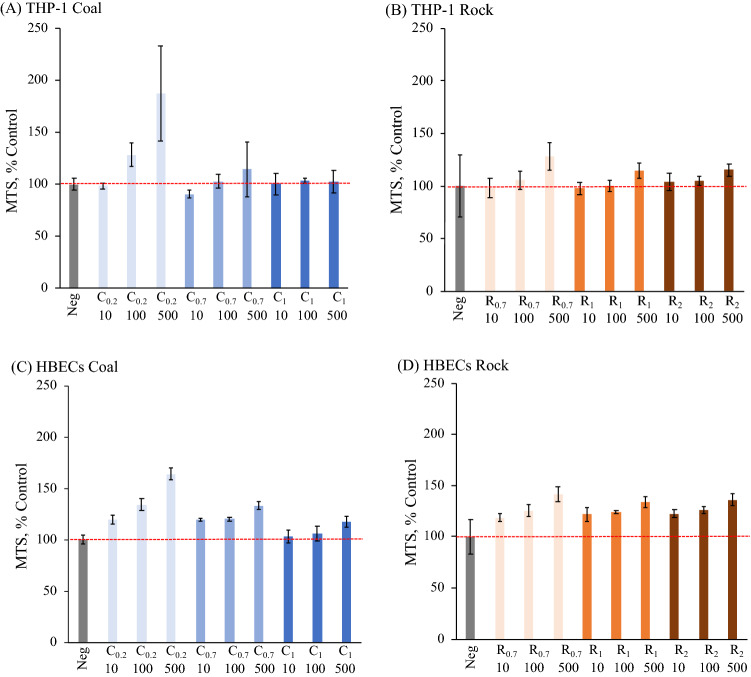
MTS analysis for (**A**,**B**) THP-1 cells and (**C**,**D**) HBECs of various coal and rock treatments (10, 100, 500 µg/ml).

**Figure 4:**
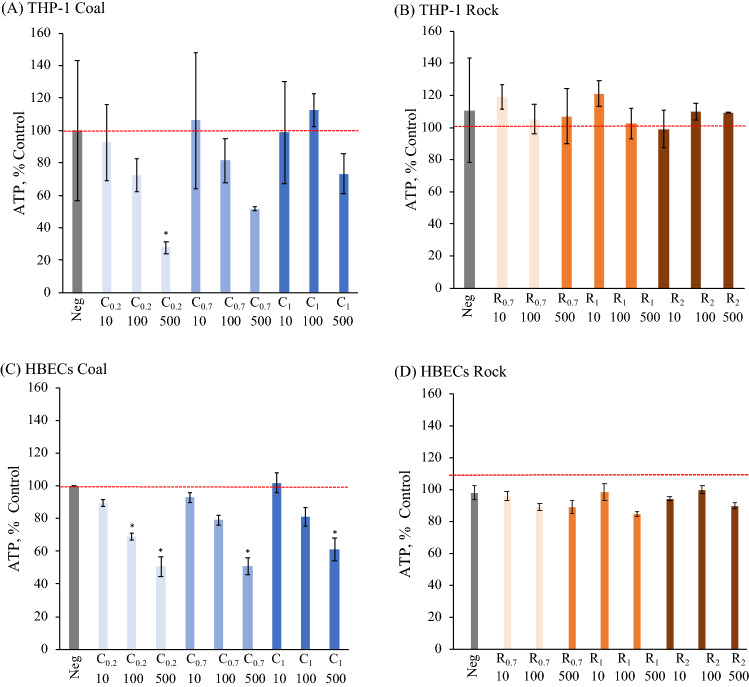
ATP analysis for (**A**,**B**) THP-1 cells and (**C**,**D**) HBECs of various coal and rock treatments (10, 100, 500 µg/ml) (*p* < 0.05*).

**Figure 5:**
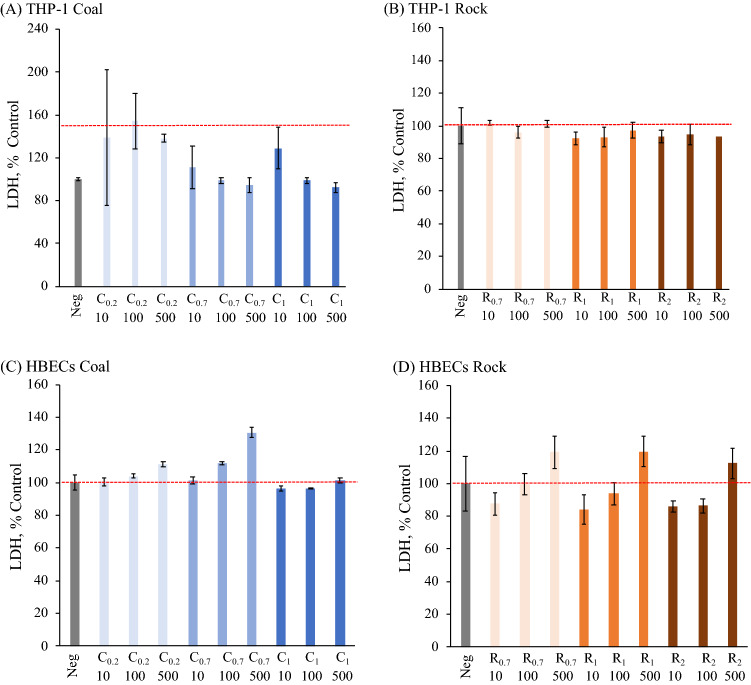
LDH analysis for (**A**,**B**) THP-1 cells and (**C**,**D**) HBECs of various coal and rock treatments (10, 100, 500 µg/ml).

The cell viability was determined by the 3-(4,5-dimethylthiazol-2-yl)-5-(3-carboxymethoxyphenyl)-2-(4-sulfophenyl)-2H-tetrazolium (MTS) assay, adenosine triphosphate (ATP) levels and lactate dehydrogenase (LDH) release is presented in Figs. 3, 4, and 5. There was no significant difference in viability as determined by MTS for THP-1 cells or HBECs treated with any rock or coal size or concentrations (Fig. 3). ATP levels displayed a decrease in coal-treated THP-1 cells and were significantly lower in the cells treated with fine coal particles at the highest concentration of 500 μg/ml (C_0.2, 500_) as compared to untreated cells (*p* < 0.05) (Fig. 4A). All 500 μg/ml treatents of coal significantly reduced ATP levels in HBEC, as well as the 100 μg/ml C_0.2_ (Fig. 4C). It is notable that none of the larger coal fractions significantly reduced HBEC ATP at a concentration as low as 100 μg/ml. The ATP levels after rock treatment of THP-1 cells and HBEC showed no significant differences. As seen in Fig. 5), LDH results with coal or rock dust treatment were similar for THP-1 cells and HBECs. LDH release, normalized relative to the extracellular LDH measured in the untreated cells’ supernatant, showed that there were no significant changes following treatment with any coal or rock processing type at any concentration.

**Figure 6:**
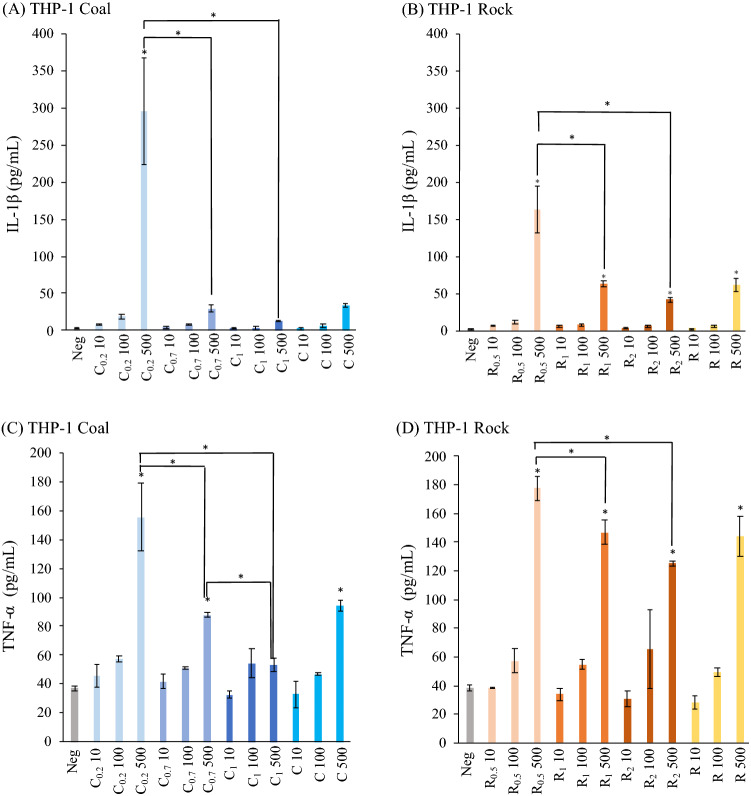
Inflammatory cytokines expressions for THP-1 cells of various coal and rock dust treatment (10, 100, 500 µg/ml): (**A**,**B**) IL-1β; (**C**,**D**) TNF-α * over an individual bar indicates p < 0.05 between the individual treatment and negative control. * over a joining bar indicates p < 0.05 between the two joined treatments.

**Figure 7:**
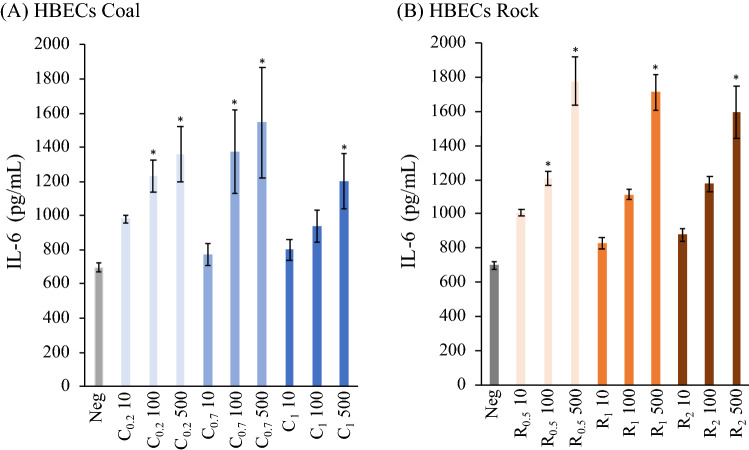
Inflammatory cytokines expressions (IL-6) for HBECs of various (**A**) coal and (**B**) rock dust treatment (10, 100, 500 µg/ml) (*p* < 0.05*).

Figures 6 and 7 show the inflammation markers measured in the culture media for THP-1 cells and HBECs following treatment with coal and rock dust. Higher cytokine release was induced as the exposure concentration increased. More interesting than the relationship of higher inflammation with concentration, is its dependence on size.

**Figure 8:**
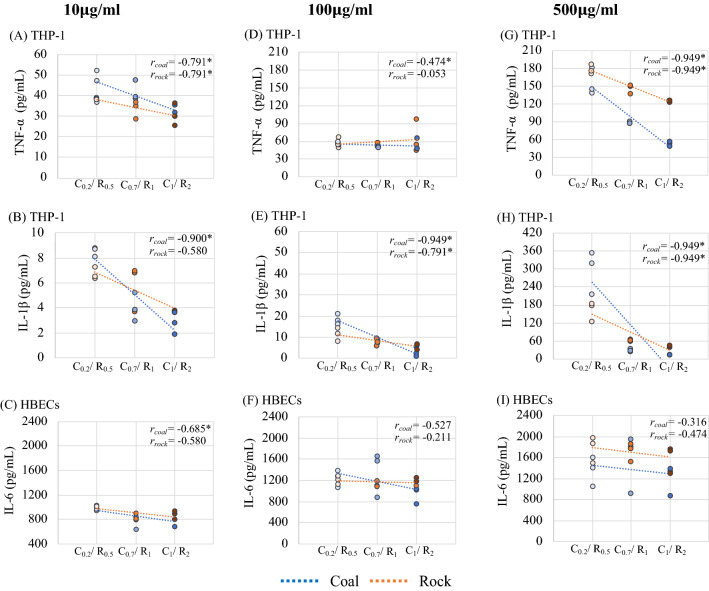
The correlation of inflammation cytokines (TNF-α, IL-1β, IL-6) between sized-mining dusts of three exposure concentration (10, 100, 500 µg/ml).

Smaller size fractions consistently induced higher cytokine release across both cell lines. THP-1 cells exposed to C_0.2_ generated significantly higher interleukin-1β (IL-1β) and TNF-α levels than the control, C_0.7_ and C_1_ groups at the highest concentration (500 μg/ml) (Fig. 6 A,C). THP-1 IL-1β levels induced by rock show the same trend: R_0.5_ induced significantly higher IL-1β and TNF-α expression than the larger-sized R_1_ and R_2_ treatments (Fig. 6B,D). In fact, when treatment concentration was controlled for, size alone was correlated with these responses. Size had a negative correlation with TNF-α expression in both rock and coal at 10 and 500 μg/ml, as well as with IL-1β in all coal treatments and 100 and 500 μg/ml rock treatments (Fig. 8 and Table S1). To summarize the size effect on inflammation, a strong negative correlation of size with inflammatory cytokines for most treatments further suggests that small particle sizes induce higher inflammation within either dust type.

Figure 7 shows that interleukin-6 (IL-6) released in HBECs culture media was significantly elevated after coal and rock treatments compared to the untreated cells, however, the expressions didn’t show a significant difference when comparing the size fractions. The smaller size was significantly associated with higher IL-6 release in coal at 10 μg/ml (Fig. 8 and Table S1). In summary, a strong size effect, given by the negative association between particle size and inflammatory response was observed in THP-1 cells compared to HBECs and is more exaggerated at higher concentrations.

## Discussion

### Fine size fraction is representative of mining dusts

A previous unpublished field sampling study concluded that miners were primarily exposed to coarse particles based on the coal particle size distribution ranging from 38 to 850 μm. However, exposures to respirable and sub-micron sized coal particles were also found. Area particle concentration measurements conducted at the coal mine field by us with a NanoScan SMPS to measure particle sizes 10–420 nm and OPS to measure 0.3–10 µm found that peak particle number count was roughly 100 nm measured by SMPS and 300 nm measured by OPS. Our separated sizes (C_0.2_ ~ 200 nm; R_0.5_ ~ 495 nm) reflect those of particles in mining dusts. Novel sampling equipment in recent studies revealed that the workers might also be exposed to nanometer-sized particles due to the use of newer technology in mining equipment creating smaller respirable particles in modern mine environments^17,26^. A survey conducted in 2007 investigated the coal dust size in a US underground mine, showing that the size of coal dust within airways was finer than the size characterized in the 1920s. The significant change of size distribution in coal dust might be related to the highly mechanized nature of modern coal mining that has increased coal production rates^20,27^. An occupational safety-based assessment reported the considerable increase in productivity was due to the introduction of new mining technology. For example, a Russian coal mining company had its productivity jump from 2270 to 7950 thousand m^3^/year. However, these economic benefits come at the cost of various health effects and safety problems due to the high amount of respirable dust particles generated. The small size fraction characterized in the present study further points to CMDLD being of continuing and growing concern, as toxicity increases with the smaller size particles and greater doses seen in modern mines. The dust particles suspensions in this present study have the tendency to aggregate, forming larger hydrodynamic diameters, despite precautions taken before exposure including sonication of the dust suspensions before treatment and vibration of the cell plate after the particle treatment. The aggregation might underestimate the cell toxicity corresponding to the size-toxicity effect.

### Particle surface hydrophobicity and hydrophilicity relate to the cell toxicity

Surface hydrophobicity and hydrophilicity can affect cell responses^28–30^. Previous studies using engineered nanoparticles have found that higher surface hydrophobicity resulted in stronger immunotoxic responses^31^. Considering the ATP assay (Fig. 4) and inflammatory cytokines (Fig. 6) in our study, the coal dust induced greater inflammation in THP-1 cells and decreased viability in both cell lines, which may be due to its surface feature difference in hydrophobicity. Several hypotheses were proposed to explain the impact of particle surface features on toxicity. Coal particles with hydrophobic surfaces aggregate more than rock particles, which can be observed from the contact angle measurement and the dispersibility in our current study, contributing to a higher size-specific deposition rate, stronger inflammation and less cell viability^32,33^. The interaction of particle surface with cell membranes was a key factor contributing to the correlation of hydrophobicity and biological interactions^34^. This correlation confirmed the positive correlation between membrane damage and nanoparticle surface hydrophobicity^35^. The hydrophobicity of coal particles might enhance the affinity for lipid membrane interactions, leading to greater cell membrane impairment than rock dust. The impairment was represented by the decrease in cell viability. The same study, conducted by Manshian et al., also indicated that the higher levels of surface hydrophobicity induced elevated autophagy leading to cell death, which can explain the greater toxicity caused by coal exposure. Coal’s increased hydrophobicity compared to rock, demonstrated by high contact angle and stronger hydrophobic functional group signal, could explain its toxicity. Notably, THP-1 cells released higher amounts of IL-1β when exposed to coal compared to when exposed to an equivalent size fraction and concentration of rock (Fig. 6A,B). A follow-up study using microscopy to identify particle cellular localization and uptake could directly confirm whether hydrophobic coal has a more deleterious effect on the lipid membrane than hydrophilic rock.

Although rock has a size-dependent effect on inflammation, the larger size fractions can still be pro-inflammatory. The magnitude of the drop in inflammatory cytokine release from R_0.5_ to R_1_/R_2_ is not as drastic as that of C_0.2_ to C_0.7_/C_1_. This may be due to rock’s mechanism of toxicity possibly relying more on chemical features rather than size, such that decreasing rock particle size only induces a small boost in inflammation. A study on lung carcinoma epithelial cells in a membrane chamber compared unmodified limestone, like the rock used in the present study, and modified limestone with a stearic acid addition to make it more hydrophobic^36^. The unmodified limestone negatively disturbed mucous viscosity, possibly by leaching Ca^2+^ ions. However, the modified limestone was less likely to leach Ca^2+^ ions, which could explain why mucus integrity was better maintained. Our rock samples share the features of relatively higher ionic strength and hydrophilicity, which may contribute to the proinflammatory effect of R_1_ and R_2_.

### Elements determine particle-induced toxicity

The elemental composition analysis detected the presence of Si, Al, Fe, Ba, Cl, K and Pt in the coal dust in our study (Table 1), which might contribute to the higher cell toxicity observed in coal compared to the rock dust particles. The relationship between elemental composition and cell toxicity has been researched, but a comprehensive understanding for the mechanism is still lacking and the precise role of individual elements in biological alterations is still debated. Si, C, and Fe detected in our coal suspension samples are known to cause pulmonary toxicity^19,37,38^. Among these elements, silica-induced toxicity has been widely discussed for decades. However, there is variability in *in-vitro* and *in-vivo* responses from numerous studies, such as reactive oxygen species generation and macrophage activation^39^. This makes it difficult to definitively identify what characteristics of silica contribute to the toxicity mechanism and clarify all interactions at the molecular level to result in lung function impairment. The present study did not directly demonstrate how hydrophobicity affects cell toxicity. However, in terms of carbon, coal’s hydrophobic nano-sized aromatic carbon particles might modify the air–liquid interfacial properties, surface tension, and behavior of pulmonary surfactants (PS) in the alveolus. The reshaping is because solubilization of phospholipids in PS increases with the degree of hydrophobicity^40^. This suggests that the particles that can penetrate through the surfactant linings induce intercellular reactions, including inflammation, oxidative stress, and cytotoxicity^40–42^.

In addition to silica, scientists have considered the role of iron. The interaction between heavy metals and PS is crucial when discussing metal-associated lung toxicity. Studies have illustrated a negative correlation between cell viability and iron content of dust in both alveolar macrophages and human lung epithelial cells^43,44^, and a systematic review summarized that iron-related genes altered mitochondrial function and levels of cytochrome oxidase, leading to mitochondrial dysfunction and the development of COPD^45^. The bioavailability of heavy metals, particularly solubility, strongly influences particle-induced pulmonary toxicity^46,47^. The leachable fraction of iron in the coal particles might potentially stimulate an increase of PS, enhancing its bioavailability and strengthening pulmonary toxicity. Interestingly, a previous biochemistry study provided evidence to support that heavy metal-rich particles might change the solubility of heavy metals in particles because of the protein component in PS^48^.

### Macrophages show a greater inflammatory response than epithelial cells on the mining dust exposure

The macrophage cells displayed a stronger inflammatory reaction than epithelial cells, suggesting that macrophage cells are more sensitive and responsive to respirable particle exposure. Different responsiveness may be due to the differing roles of HBECs and macrophages. Active phagocytosis of particles by macrophages likely allows for more uptake and direct particle interaction. Although epithelial cells are involved in the inflammatory response and uptake cells, they function more passively in comparison and have relatively lower active uptake. This could explain the observable sensitivity difference in cellular response. Another hypothesis relates to the cell locations. As an endpoint of the respiratory system, alveolar macrophages will intake the particle for clearance and release cytokines or mediators to signal the bronchiolar epithelial cells. When the intake particles accumulate, macrophages would be the first place to trigger the toxicity and poison the following sites^49^. The release of the inflammatory endpoints, even the formation of pulmonary disease, involves complicated interactions between various cells, tissues, and mediators. Once the respirable coal or rock dust particles enter the body, macrophages are activated and secrete numerous pro-fibrotic soluble mediators, cytokines, and matrix metalloproteases and serve as crucial regulators of fibrosis^50–52^. If the stimulative process is not impaired via normal molecular interactions, it leads to the damage of epithelial cells and lung tissue injury^53^. The understanding of the lung macrophages’ role in the fibrotic process was expanded recently when toxicologists found that macrophages can adjust their functional phenotype to adapt to various environments. This complicates the research of the macrophage role in pulmonary disease development and particle-induced lung toxicity mechanism^51^, particularly as it is related to particles with different physicochemical characteristics.

### Significance

**Figure 9:**
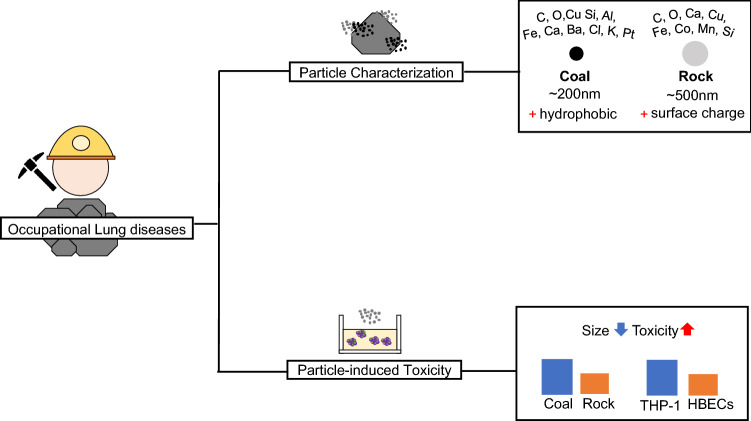
Summary for present study: the dependence of particle size on cell toxicity for modern mining dust.

Figure 9 shows the summary for the present study. The significance of this study include: the exploration of particle size effect and related physio-chemical characterization of respirable ground modern mining dusts with molecular toxicity that can be utilized in future lung disease risk reassessment. Additionally, the particle characterization results in this study emphasize the importance of dust properties in different dust exposures in particle-induced cell reactions. Although this study found no significant differences in pulmonary cell viability, the difference in inflammatory endpoints induced by dust particles is still explored in the in-vitro study. This is consistent with toxicity studies showing marginal differences in viability despite substantial stress responses^44^, as well as epidemiological evidence of sustained stress response and disease.

Several suggestions can be applied in future studies to elucidate the molecular mechanism causing occupational pulmonary toxicity and determine a dose–response correlation for the respirable mining dusts in the modern mine workplace. Future studies can extend the treatment duration and increase the exposure concentration to better resemble the reality of workplace mine dust exposure. Considering the *in-vitro* study, this present study utilized a two-dimensional cell culture to investigate the cell response. Although it is useful to initially explore the relationship between particle exposure and cell reactions, a three-dimensional cell culture with an air–liquid interface has been indicated as a more appropriate environment that can imitate the human respiratory system^54,55^. Additional pulmonary toxicity endpoints can be examined, such as mitochondrial function, intercellular uptake, and oxidative stress to explore more cell reactions, which might be beneficial to establish a reliable pathway and model the dose dependency of the effects. The actual intracellular dose and localization were unclear after treatment with dust suspensions for 24 h. Further studies should apply bio-TEM or histological stains like hematoxylin and eosin stain (H&E stain), to identify the particles entering through the cell membranes and their behavior inside the cells.

### Conclusions

The present study explored the finer fraction of approximately 200 nm in coal dust particles from an underground modern mine ground in the laboratory. In-vitro findings support that fine particle fractions explicitly induced stronger toxicity than their coarser sizes in coal dusts. The reaction difference between coal and rock might be related to surface hydrophobicity and elemental composition. Macrophages are more sensitive to particle-induced toxicity than epithelial cells. In summary, this occupational-based research explored the impact of particle physicochemical characterizations on dust-induced pulmonary toxicity. Further studies should be conducted to elucidate the nanotoxicity mechanism that causes occupational pulmonary diseases and determine the exposure–response correlation of respirable mining dusts and cell responses.

## Material and methods

### Study design

This study used in-vitro cell models with particle characterization including size, morphology, surface hydrophobicity and elemental compositions to evaluate the effects of finer size fractions in mining dust and explore the potential contributing particle characteristics to cell toxicity. Raw coal and rock dust from the underground mine were characterized to determine their physicochemical properties and elemental compositions. The in-vitro study with two cell lines, human THP-1 and HBEC, modeled macrophage reaction and epithelial damage. The rock and coal particle suspensions were separated following the method detailed in section "Surface features of raw coal and rock dust". The cells were exposed to three different sizes of rock and coal dust obtained from the top, middle, and bottom layer of the suspension, representing small, medium, and large, respectively, at concentrations of 10, 100 and 500 μg/ml. Dependent toxicity levels were determined via cell viability and inflammation responses.

### Material and dust separation

The raw coal and mine rock dust from the mine site (Colorado, USA) were ground by using a Sepor Bond Ball Mill (Sepor, Inc, CA, USA) with standard carbon steel balls for approximately 60 min and sieved by a 400 mesh (~ 37 µm) on a vibratory plate in a laboratory in October 2019. The dust samples were then separated into three size ranges (100–500 nm, 500–1000 nm, > 1000 nm) using a density gradient centrifugation method with solutions of 10, 20 and 40% w/v polyethylene glycol (PEG, 20 K) in water where larger particles settled toward the bottom and smaller particles toward the top. The centrifugation procedure differed for coal and rock due to their deposition. For coal, aliquots of 10 mL of 20% w/v PEG solution were carefully layered on top of the 40% w/v PEG solution within a 50 mL centrifuge tube. Next, 10 mL of the solution containing 18 mg/mL coal dust solution was dropped at the top of the PEG layers. The coal tube was centrifuged at 100 RCF for 1 min, resulting in two distinct layers. For rock, the gradient of 10%, 20%, and 40% w/v PEG solution was created. A 50 mg/mL rock dust solution was dropped at the top of the PEG layers. Following, the rock tube was allowed to settle for 10 min, resulting in three distinct layers. Mixing layers was avoided during the process.

After centrifugation or deposition, 3 size ranges were isolated. For coal, 1 mL of solution from the distinct top and bottom layers was collected, as well as 1 mL from the boundary between the layers to be the middle layer. Despite only having two layers, particles obtained from the boundary provided a reproducibly distinct size fraction confirmable by DLS and TEM, resulting in three size ranges. For rock, 1 mL of solution from the top, middle, and bottom layers was collected. The top layer contained the smallest size range, the middle layer contained an intermediate size, and the bottom layer contained the largest. The dust in the PEG solution was washed twice with water to remove PEG (10,000 RCF, 20 min). The washed dust samples were dried overnight in a vacuum oven at 50 °C. The dry samples were weighed and then suspended in water (2 mg/mL) to be analyzed by DLS and TEM. Separated dust particle samples were named based on the size ranges from the DLS measurement and the TEM observation.

### Dust characterization

Physicochemical properties including particle size range, morphology, surface charge and hydrophobicity, surface functional groups and elemental compositions were analyzed in coal and rock.

#### Physicochemical properties

After size separation, the dust samples were dropped in a cuvette and measured by DLS to determine size distributions. The morphology of samples was examined and DLS sizing was confirmed by TEM (FEI Technai T12 electron microscope, 120 kV, Point resolution: 0.34 nm, Line resolution: 0.2 nm). A ZetaPALS analyzer and a contact angle goniometer examined surface charge and hydrophobicity. The sample preparation for the ZetaPALS analyzer was the same as DLS analysis. For contact angle measurement, dust samples were packed into a hydraulic pellet press to make a surface. 300 mg of dust samples were pressed at 7000 psi for 3 min. Immediate contact angle with water was taken within 1 s and averaged for 3 pellets.

#### Composition analysis

Dust suspensions of 2 mg/mL concentration were vortexed and sonicated for 15 s, then a drop of the suspension was put on a Quantifoil ™ R 2/1 on a 200 mesh TEM copper grid and dried. STEM w/ EDX (FEI Titan S/TEM electron microscope, Oxford X-Max TEM EDS system, 80 kV) detected the elemental composition of the sample on the TEM grid, subtracting a background of an empty identical grid.

Functional groups were determined using FTIR (Agilent Cary 670 FTIR Spectrometer, Agilent Technologies, CA, USA). A background reference was obtained with an empty cartridge. 2 mg of dust and 200 mg of KBr were ground together, packed into a pellet press and pressed at 10,000 psi for 10 min. The spectral range was 4000–400 cm^−1^, the resolution was 4 cm^−1^, and each sample was scanned 64 times. Spectra of absorbance to wavenumber were deconvoluted and baseline corrected. Peak area of functional groups was taken for hydroxyl structures in 3800–3000 cm^−1^, aliphatic structures in 3000–2800 cm^−1^, oxygen-containing functional groups in 1800–1000 cm^−1^ and the aromatic structures in 900–700 cm − ^1^ from absorbance spectra^56^.

### Cell culture and particle exposure

Human THP-1 cells from ATCC (Manassas, VA, USA) were grown in RPMI-1640 media with 10% fetal bovine serum (FBS), 100 U/100 μg per mL of penicillin–streptomycin, and 50 × 10^–6^ mol/L beta-mercaptoethanol. HBECs obtained from American Type Culture Collection (ATCC, Manassas, VA) were cultured in supplemented BronchiaLife epithelial airway media as described by Lifeline technologies (Lifeline Cell Technology, Frederick, MD).

The Thermo Scientific™ Pierce™ Chromogenic Endotoxin Quant Kit ensured no endotoxin contamination in the unseparated dust powder before applying the *in-vitro* treatment. Coal and rock dust particulates from the top, middle and bottom separations (described in 2.2) were weighed out and resuspended in culture media to a concentration of 10, 100, and 500 μg/mL. THP-1 cells were plated at 3–5 × 10^4^ cells per well and HBECs were plated at 1.1 × 10^4^ cells per well into 96 well plates. After incubating overnight at 37 °C in a 5% CO_2_ incubator, cells were treated with various coal or rock samples in triplicates for 24 h.

Concentrations of 10, 100, and 500 μg/mL were selected to represent a range of viability and inflammatory responses based on previous sampling and in-vitro experimentation. The average breathing zone mass concentrations from previous coal mine sampling (unpublished data) were measured to be around 1.21 mg/m^3^ (0.00121 μg/mL) for Tsai diffusion sampler (TDS) and 1.16 mg/m^3^ (0.00116 μg/mL) for polyvinyl chloride (PVC) membrane filter sampling. Roughly 8–10 in-vitro experiments of concentrations ranging from 1 to 800 μg/mL were performed. These preliminary experiments showed minimal effects under 500 μg/mL and massive cell death over 500 μg/mL, which showed little promise in elucidating inflammatory dose–response. Although the exposure concentration is higher than actual exposures, it is still beneficial in determining the effect of physicochemical properties on nanotoxicity.

### Cytotoxicity measurements

Cell toxicity was determined via cell viability and inflammatory cytokine production. Cell survival rate was measured with MTS (inner salt), ATP, and LDH for both cell lines.

1.5 mL MTS dye (CellTiter 96 Aqueous, Promega Corp) in 7.5 mL cell culture medium were prepared to be MTS working solutions for each plate. The supernatants were aspired and transferred to new 96 well plates for inflammation markers analysis. 100 μL of the working solution was added to the cells per well and was incubated for 15–20 min at 37 °C with a humidified 5% CO_2_. Formazan absorbance was read at 490 nm on a SpectraMax M5 microplate reader (Molecular Devices Corp., Sunnyvale, CA, USA).

ATP levels were analyzed after 24 h exposure with the ATPlite™ 1-step assay kit (PerkinElmer, Inc., Waltham, MA, USA). 100 μL of ATPlite 1step reagent was added to the remaining cells per well. The resulting luminescence was monitored at 500 nm on the SpectraMax M5 microplate reader. CytoTox 96® Non-Radioactive Cytotoxicity Assay measured LDH expression (CytoTox 96® Non-Radioactive Cytotoxicity Assay, Promega). Cells were incubated for 24 h. and then spun down to remove the supernatant to measure LDH release. Researchers followed the protocol provided by Promega after obtaining supernatant and the absorbance was measured using a fluorescent spectrophotometer at 490 nm (BioTek Synergy HT, BioTek, Winooski, VT; software: Gen5 2.04). All the MTS, ATP, and LDH values of treated cells were normalized according to the MTS, ATP, or LDH measured in non‐treated control, which exhibited ~ 100% cell viability.

### Inflammation markers

Cytokine expression of TNF-α, IL-1β, and IL-6 in cell culture media supernatant, which reflects inflammatory response, was examined after various rock and coal dust exposure by enzyme-linked immunosorbent assay (ELISA). The commercial kits for ELISA were performed following the manufacturer’s protocols (Thermo Fisher Scientific, Inc., Waltham, MA, USA) (IL-6 Human IL-6 DuoSet ELISA, R&D Systems, Minneapolis, MN, USA).

### Statistics

Data is presented as the mean ± standard error mean (SEM). One-way or two-way analysis of variance (ANOVA) with Bonferroni post-hoc testing was utilized to test for significant differences among multiple exposure groups. Pearson’s correlation coefficient was used to determine correlation between ranked size and inflammatory markers. Concentration and dust type were controlled for in order to isolate the correlation with size. T-test was utilized where applicable to test for significant differences between two exposure groups. A *p* value of < 0.05 was considered statistically significant.

## Supplementary Information

Supplementary Information.

## Data Availability

The data used to support the finding of this study are included within the article.
