# Supplementary material for: The dependence of particle size on cell toxicity for modern mining dust
Source: Sci Rep. 2023 Mar 29;13:5101. doi: 10.1038/s41598-023-31215-5 (PMC10060429; doi:10.1038/s41598-023-31215-5)
Supplement: Supplementary file 1 — Supplementary Information. [file 41598_2023_31215_MOESM1_ESM.docx]

**The dependence of particle size on cell toxicity for modern mining dust**

**Supplementary Information**

Table S1. Pearson’s correlation between inflammation cytokines (TNF-α, IL-1β, and IL-6) and size, controlled for concentrations (10, 100, 500 µg/ml) and dust type. The mining dusts are ranked as follows in the analysis: the smallest size of dust particles (C_0.2_ and R_0.5_)=1, the middle size of dust particles (C_0.7_ and R_1_) =2, and the biggest size of dust particles (C_1_ and R_2_).

| Concentration (µg/ml) | Mining dust | THP-1 TNF-α | | |  | | THP-1 IL-1β | | |  | | HBECs IL-6 | |  |
| --- | --- | --- | --- | --- | --- | --- | --- | --- | --- | --- | --- | --- | --- | --- |
|  |  | Coefficient, r | *p*-Value |  | | Coefficient, r | | *p*-Value |  | | Coefficient, r | | *p*-Value | |
| 10 | Coal | -0.791 | 0.011^*^ |  | | -0.900 | | 0.0011^*^ |  | | -0.685 | | 0.042^*^ | |
|  | Rock | -0.791 | 0.011^*^ |  | | -0.580 | | 0.1322 |  | | -0.580 | | 0.102 | |
| 100 | Coal | -0.474 | 0.197 |  | | -0.949 | | 0.0001^***^ |  | | -0.527 | | 0.145 | |
|  | Rock | -0.053 | 0.893 |  | | -0.791 | | 0.0112^*^ |  | | -0.211 | | 0.586 | |
| 500 | Coal | -0.949 | 0.0001^***^ |  | | -0.949 | | 0.0001^***^ |  | | -0.316 | | 0.407 | |
|  | Rock | -0.949 | 0.0001^***^ |  | | -0.949 | | 0.0001^***^ |  | | -0.474 | | 0.197 | |

^*^ *p<* 0.05; ^**^ *p<*0.01; ^***^ *p<*0.0001

Figure S1. The dispersibility of 1 mg/ml (A) Rock; and (B) Coal suspensions. 2 mg/mL size-separated coal in water solution following dispersion by vortex. (C) C_0.2_, (D) C_0.7_, and (E) C_1_ represent sizes of fine, mixed, and coarse coal particles from top, middle, and bottom layers after separation.

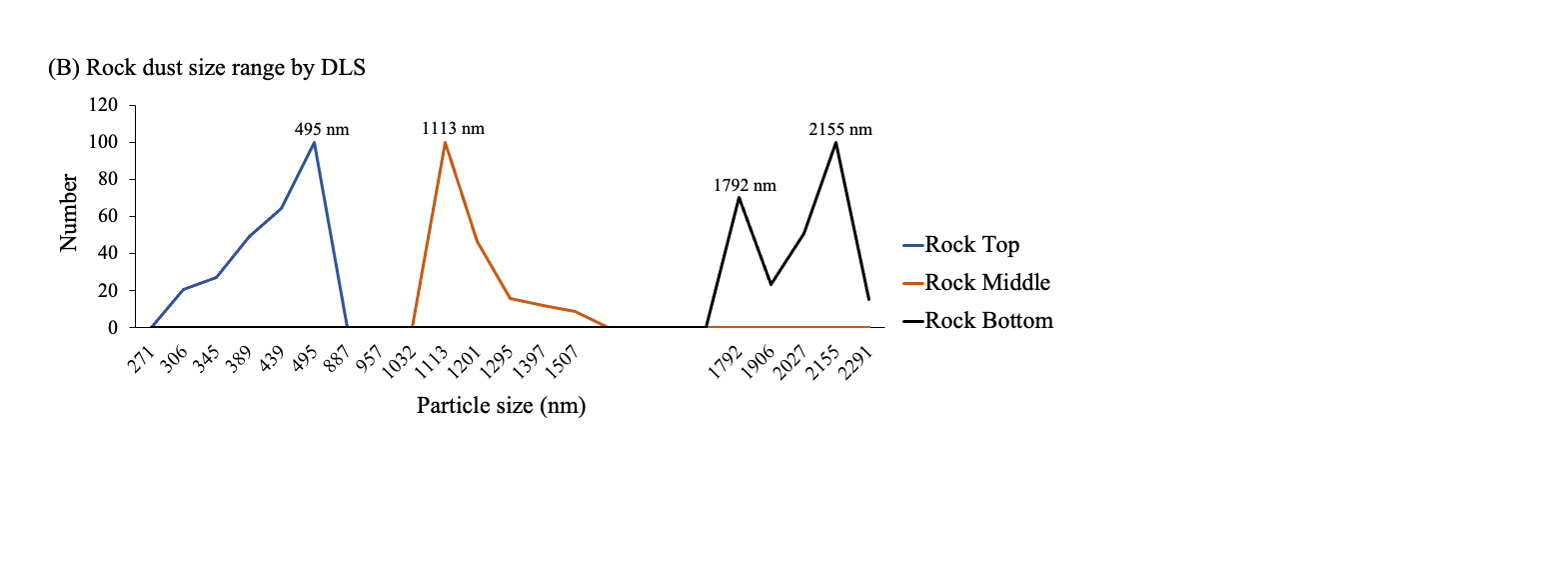


Figure S2. Hydrodynamic size by count of (A) Coal and (B) Rock samples in water from top, middle, and bottom layers. Top layer has a mix of small and large particle, indicated by two size peaks, but generally has more of a small particle fraction (~200 nm) than the middle and bottom layers. *Bottom layer size was determined by TEM because of the measurement limitation by DLS cuvette.

Figure S3. (A) Immediate contact angle of coal dust was 86° ± 5 averaged for 3 pellets; (B) Water contact angle for rock pellets was immediately dispersed.

(A)


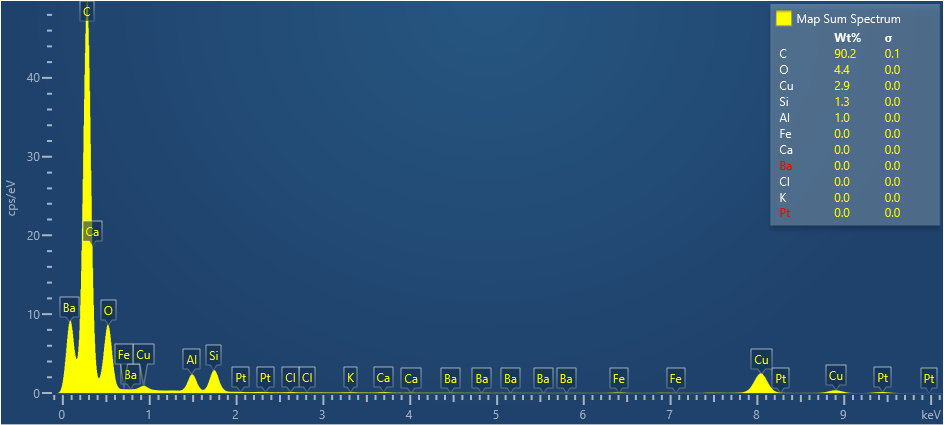


(B)


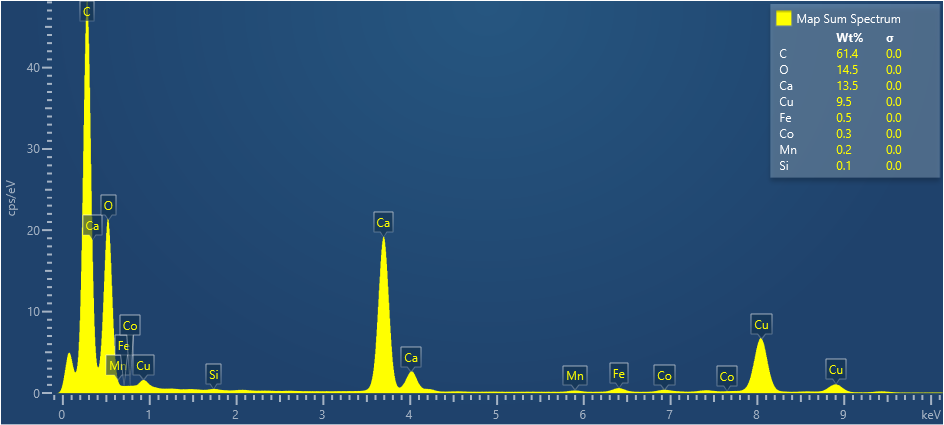


Figure S4**.** The spectrum map from STEM w/ EDX indicates the element compositions for (A) Coal and (B) Rock dust particles.


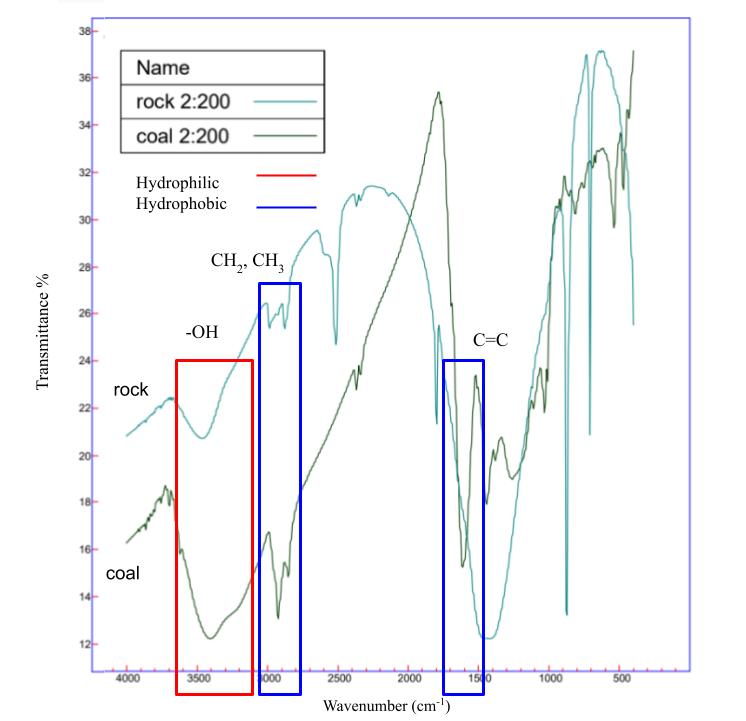


Figure S5. FTIR spectra of coal showed that it has a relatively stronger aliphatic signal compared to its hydroxyl signal, as well as a C=C bend that is not present in rock. This suggests that coal contains more C-containing groups than O-containing groups which contribute to its hydrophobicity.
